# Transcriptomic Analysis of Mouse Cochlear Supporting Cell Maturation Reveals Large-Scale Changes in Notch Responsiveness Prior to the Onset of Hearing

**DOI:** 10.1371/journal.pone.0167286

**Published:** 2016-12-05

**Authors:** Juan C. Maass, Rende Gu, Tiantian Cai, Ying-Wooi Wan, Silvia C. Cantellano, Joanna S. T. Asprer, Hongyuan Zhang, Hsin-I Jen, Renée K. Edlund, Zhandong Liu, Andrew K. Groves

**Affiliations:** 1 Department of Neuroscience, Baylor College of Medicine, 1 Baylor Plaza, Houston, TX, United States of America; 2 Program in Developmental Biology, Baylor College of Medicine, 1 Baylor Plaza, Houston, TX, United States of America; 3 Department of Molecular and Human Genetics, Baylor College of Medicine, 1 Baylor Plaza, Houston, TX, United States of America; 4 Department of Pediatrics, Baylor College of Medicine, 1 Baylor Plaza, Houston, TX, United States of America; 5 The Jan and Dan Duncan Neurological Research Institute, Texas Children’s Hospital, Houston, TX, United States of America; 6 Department of Otolaryngology, Hospital Clínico Universidad de Chile and Interdisciplinary Program of Physiology and Biophysics ICBM Universidad de Chile, Santiago, Chile; 7 Department of Otolaryngology, Clínica Alemana de Santiago, Facultad de Medicina Clínica Alemana-Universidad del Desarrollo, Santiago, Chile; University of Washington, UNITED STATES

## Abstract

Neonatal mouse cochlear supporting cells have a limited ability to divide and trans-differentiate into hair cells, but this ability declines rapidly in the two weeks after birth. This decline is concomitant with the morphological and functional maturation of the organ of Corti prior to the onset of hearing. However, despite this association between maturation and loss of regenerative potential, little is known of the molecular changes that underlie these events. To identify these changes, we used RNA-seq to generate transcriptional profiles of purified cochlear supporting cells from 1- and 6-day-old mice. We found many significant changes in gene expression during this period, many of which were related to regulation of proliferation, differentiation of inner ear components and the maturation of the organ of Corti prior to the onset of hearing. One example of a change in regenerative potential of supporting cells is their robust production of hair cells in response to a blockade of the Notch signaling pathway at the time of birth, but a complete lack of response to such blockade just a few days later. By comparing our supporting cell transcriptomes to those of supporting cells cultured in the presence of Notch pathway inhibitors, we show that the transcriptional response to Notch blockade disappears almost completely in the first postnatal week. Our results offer some of the first molecular insights into the failure of hair cell regeneration in the mammalian cochlea.

## Introduction

The death of auditory hair cells due to noise damage, ototoxins or aging is a principal cause of sensorineural hearing loss [1–3]. In contrast to other vertebrates, where supporting cells readily re-enter the cell cycle and generate hair cells after damage, the mature organ of Corti is unable to regenerate [1, 4–7]. However, recent studies suggest that neonatal mouse supporting cells retain a limited, transient capacity for regeneration. For example, neonatal mouse supporting cells are able to down-regulate cell cycle inhibitors, re-enter the cell cycle and generate hair cells in culture [8–10]. This cell cycle re-entry can be driven by activation of the Wnt signaling pathway [11–15] or by deletion of cell cycle regulators such as *p27*^*Kip1*^ [16]. Blockade of Notch signaling between hair cells and supporting cells can result in trans-differentiation of supporting cells into hair cells [13, 17–20]. Such trans-differentiation of supporting cells can also be observed at very low levels after hair cell killing [17, 21]. Finally, ectopic activation of the hair cell-specific transcription factor *Atoh1* in supporting cells can drive their differentiation into hair cells [12, 22–24]. In all these cases, however, the capacity of mouse supporting cells to either divide or trans-differentiate into hair cells is lost between birth and the onset of hearing at two weeks of age [1, 9, 22, 23, 25].

All supporting cells in the mouse organ of Corti are generated prior to birth and undergo dramatic morphological changes, such as the elaboration of phalangeal processes, and formation of the reticular lamina and the tunnel of Corti [4, 26, 27]. This functional maturation of supporting cells, together with the decline in their regenerative ability over the first two weeks of postnatal life is likely to be reflected by transcriptional or epigenetic changes. To better understand the molecular basis for these changes, we performed an RNA-seq-based analysis of purified cochlear supporting cells from 1- and 6-day old mice. We find large scale gene expression changes consistent with morphological maturation including changes in the cytoskeleton and the extracellular matrix, together with changes in the gene regulatory network over this time period.

We and others have demonstrated that the ability of supporting cells to trans-differentiate into hair cells after Notch inhibition declines dramatically in the first postnatal week [19]. To understand this phenomenon, we performed RNA-seq analysis of purified supporting cells from new born (P0)-or 5-day old cochleas (P5) that had been cultured for 24 hours in the presence of the Notch inhibitor DAPT. Strikingly, we found that while over 2,000 transcripts were significantly altered as P0 supporting cells trans-differentiated into hair cells, only 20 transcripts changed significantly in P5 cochleas cultured in the same conditions. Our study has identified the transcriptional signature of supporting cell maturation and shows that the Notch pathway is greatly attenuated during the first postnatal week.

## Materials and Methods

### Experimental animals

*Lfng*^*EGFP*^ mice (Tg*(Lfng-EGFP)*HM340Gsat) were generated by the GENSAT project [28–30] and obtained from Dr. Nathaniel Heintz. Neonatal mouse pups were taken for organ cultures and cell sorting at postnatal day (P) 0, P1, P5 and P6. ICR or CF1 mice were used for in situ hybridization and for generating RNA from whole cochleas. The Baylor College of Medicine Institutional Animal Care and Use committee and the Faculty of Medicine of Universidad de Chile Bioethics committee approved all animal experiments. Genotyping for the *Lfng*^*EGFP*^ transgene was performed with primers to GFP (Forward primer: CGA AGG CTA CGT CCA GGA GCG CAC; Reverse primer GCA CGG GGC CGT CGC CGA TGG GGG TGT, yielding a 300bp band.

### Cochlear isolation and culture

P0, P1, P5 and P6 cochlear explants were dissected and cultured as previously described [19]. Briefly, following euthanasia, mouse heads were bisected, the temporal bone was removed from the skull base and the otic capsule was removed with forceps to separate the intact membranous cochlea from the surrounding bony structures. For P0 and P1 animals, the cochlear duct was peeled away from the modiolus and the medial structures (Kölliker’s and Corti´s organs) were separated from the lateral wall, Reissner´s membrane and the stria vascularis. For P5 and P6 mice, the cochlear duct was gently separated from the modiolus by cutting between them with forceps to preserve the organ of Corti. The lateral wall, stria vascularis and Reissner´s membrane were then partially removed by cutting with a 27 gauge needle. P0 and P5 cochleas were cultured in DMEM/F12 (buffered with Hepes; Thermo Fisher Scientific) supplemented with B27 (Thermo Fisher Scientific), 1mM N-acetylcysteine (Sigma), 5 ng/ml EGF and 2.5 ng/ml FGF2 and 67 μg/ml penicillin. For Notch inhibition experiments, cultures were treated with 10μM DAPT (Gamma secretase inhibitor IX, Calbiochem EMD) or 0.04% v/v DMSO (vehicle control; Thermo Fisher Scientific) for 24 hours as previously described [19].

### Immunostaining

Mid-modiolar 14μm frozen sections of paraformaldehyde-fixed temporal bones of *Lfng*^*EGFP*^ mice were washed, permeabilized and blocked in PBS containing 0.2% Triton X-100 and 10% donkey serum. They were then incubated with primary antibodies, washed in PBST (0.1% Triton X-100 in PBS) and incubated with secondary antibodies, followed by 3 washes in PBST. Primary antibodies used were rabbit polyclonal anti-MYOSIN VI (1:500; Proteus Biosciences), anti-GLAST (1:500; Abcam), anti-PROX1 (1:1000; EMD Millipore), anti-SOX2 (1:250; EMD Millipore) and anti-p75^NTR^ (NGFR: 1:200; Advanced Targeting Systems AB- N01 AP). Secondary antibodies were Alexa Fluor 594 (Invitrogen). Images were obtained on an Axio Observer Zeiss microscope with an Apotome2 structured illumination attachment and analyzed in Axiovision 4.8 (Zeiss).

### Cell Dissociation and FACS sorting

For supporting cell sorting, explants were washed in ice cold Ca^2+^, Mg^2+^-free -PBS and incubated at 37°C in EBSS solution containing papain (20U/ml), 1mM L-cysteine and 0.5mM EDTA (Worthington, Lakewood, NJ) for 8 minutes (P0 cultured explants) or 10 minutes (all other organs). The papain solution was removed and inactivated by gentle washes in ice cold PBS containing 2% FBS. The cells were dissociated by gently pipetting on ice and filtered with a cell strainer cap (BD Biosciences). The dissociated and filtered cells were sorted in a FACSAriaII cell sorter (BD Biosciences) at 4°C in PBS containing 2% FBS, using a 130μm nozzle. The cells were collected on the basis of their fluorescence gating in DMEM 5% FBS, spun down, lysed in RTL buffer (Qiagen) and stored at -80°C. For each RNAseq library, approximately 50,000 sorted cells were used from the freshly dissected cochleas, and 10,000 sorted cells were used from the cultured explants. The identity of sorted cells was confirmed using epifluorescence and qRT-PCR for hair cell and supporting cell markers. Duplicates samples were collected for each condition.

### RNA Extraction

For RNASeq libraries, total RNA was extracted using Trizol (Thermo Fisher Scientific) and PelletPaint (Novagen EMD Millipore). Briefly, samples were homogenized in 300μl of Trizol and shaken with 60μl chloroform and 2μl PelletPaint and centrifuged for 15 minutes. The supernatant containing RNA was precipitated with 1 volume of isopropanol, centrifuged for 30 minutes, washed in 75% ethanol, resuspended in 100μl water and re-precipitated with 10μl 3M sodium acetate and 250μl ethanol. After a final wash in 75% ethanol, the pellet was allowed to dry, resuspended in 20μl RNAse-free water and stored at -80°C. For qRT-PCR, RNA was prepared using an RNeasy Micro kit (Qiagen).

### RNA probe synthesis

Primer sets for each candidate gene were selected to target a 500–700 bp DNA fragment in a single exon of each gene for screen. A T7 RNA polymerase sequence (5’-GGATCCTAATACGACTCACTATAGGGAG-3’) was added to the 5’ end of each reverse primer. Primer sets used are listed in S1 Table. Mouse genomic DNA was used as the template for PCR. The PCR product of the correct size was purified with a PCR Purification Kit (Qiagen). Purified DNA was used as the template for RNA probe synthesis with T7 polymerase (Promega) using standard protocols [31].

### In situ hybridization

The *in situ* hybridization procedure for frozen sections was carried out as recently described [19], and for whole mounts as described in [32]. A probe concentration of 1μg/ml was used for sections and 0.3–0.8μg/ml for whole mounts.

### qRT-PCR

cDNA was prepared using approximately 100–500ng of total RNA, random primers and the SuperScript III First-Strand Synthesis System (Invitrogen). qPCR reactions were performed with SYBRGreen PCR Master Mix in a StepOne Plus Real Time PCR machine (Applied Biosystems), using in the reaction at 0.3 to 0.6 ng/μl cDNA and 50nM primers. Primers sequences used in this study were: *Myo6*: Forward primer—5’ -TGTTAAGGCAGGTTCCTTGAAG-3’, Reverse primer—5’ -ACACCAGCTACAACTCGAAAC-3’, *Prox1*: Forward primer—5’-CGTTACGGGAGTTTTTCAATG-3’, Reverse primer—5’-CCTTGTAAATGGCCTTCTTCCA-3’, *Gapdh*: Forward primer—5’-AGGTCGGTGTGAACGGATTTG-3’, Reverse primer—5’-TGTAGACCATGTAGTTGAGGTCA-3’. *Gapdh* was used as reference gene. Statistical analysis of qRT-PCR data was performed with a Mann-Whitney test using PAST statistical software.

### RNA sequencing

Duplicate samples of GFP+ and GFP- sorted cells were prepared for each experimental condition. 10,000–60,000 sorted cells were used as starting material to generate approximately 100–600 ng RNA, as measured by a Nanodrop spectrophotometer. cDNA libraries for RNAseq were generated using RNA Seq Truseq RNA sample preparation kit v2 (Illumina) following the “low sample” protocol according to the manufacturer's instructions for mRNA extraction, cDNA synthesis, indexing and amplification. cDNA generated from less than 20,000 cells received an initial amplification using the NuGen Ovation Kit. The quality and integrity of RNA samples and the final quality of the sequencing libraries was checked by electrophenogram in an Agilent Bioanalyzer. Paired-end sequencing was performed in HiSeq2000 sequencing platform (Illumina). Fastq files of paired end reads have been deposited in the NCBI GEO database, Accession No. GSE83357.

### Bioinformatic analysis

Analysis of the sequencing reads was performed by two different approaches. (1) Reads were mapped to the Mus musculus NCBI build37.2 iGenome (Ilumina) using TopHat 2.0 software [33, 34] and the mapped reads were quantitated and compared using Cufflinks 2.0 providing differential gene expression data and statistics. (2) Reads were aligned to the Mus musculus Ensembl mm9 iGenome (Ilumina) using TopHat 1.4.1 software and the number of reads per gene and per library was obtained using DESeq program. From the 24 cDNA libraries (12 duplicates) 50–200 million of paired end reads were obtained from each library. Of those reads 86 to 99% were correctly mapped and 73 to 93% were properly paired. After comparing the level of expression of each gene within each pair of related libraries, the most significant differentially expressed genes (DEG) were annotated and analyzed separately for both approaches. In order to find enriched genes in freshly isolated Lfng-EGFP^+^ cells, a significantly DEG was considered to have an RPKM higher than 3000, Fold Change (FC) higher than 4 and *p* value and FDR < 0.01. The level of DEG significance for comparing cultured explants (treated or not with DAPT) sample libraries was a FC higher than 2 and a *p* value and FDR < 0.01. To evaluate our Notch inhibition experiments where cultures were treated with DAPT or DMSO (in which supporting cells may trans-differentiate into hair cells), we used our consensus gene list of P1 Lfng-EGFP^+^ cells to represent supporting cells. This list was used to identify supporting cell genes down-regulated by DAPT treatment. All the complete transcriptomes obtained in this study have been included in S2 and S3 Tables.

### Gene Ontology (GO) analysis

The lists of differentially expressed genes in each experiment were uploaded into the DAVID bioinformatics suite [35–38] for gene ontology analysis. We obtained GO terms related to biological processes, and the long list of terms was uploaded to REVIGO [39] using default parameters to summarize it in representative terms. The threshold to detect redundancies used was set arbitrarily to 0.1. The REVIGO output was complemented with DAVID output in order to create merged tables. In all tables only represented terms with p<0.01 were included.

## Results

### RNA-seq analysis identifies mouse supporting cell transcripts differentially expressed in the first postnatal week

**Fig 1:**
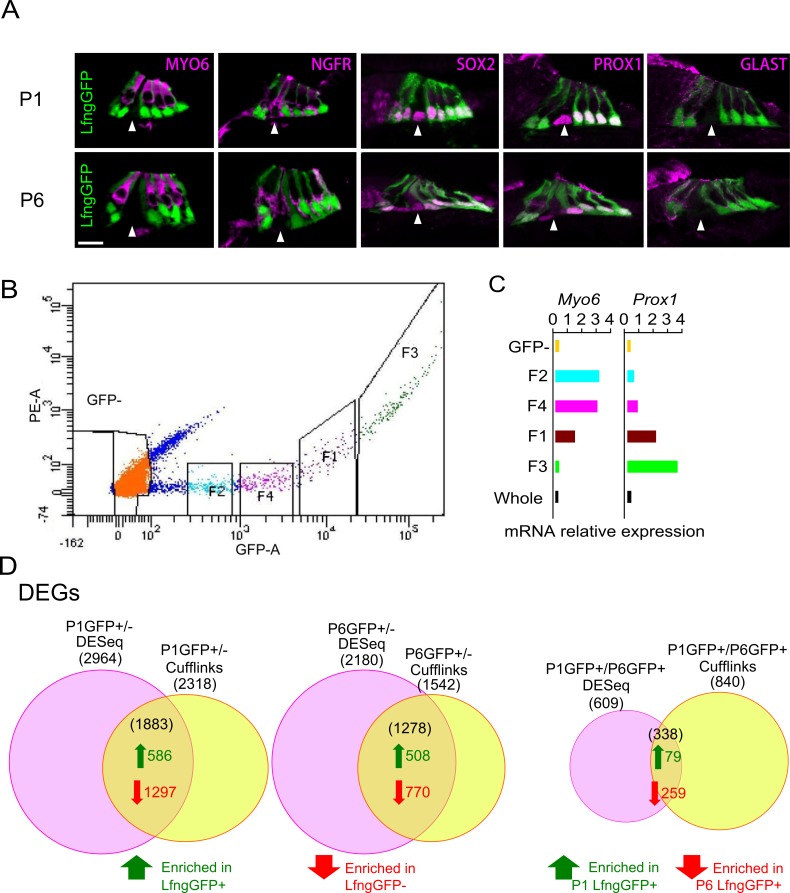
Isolation of neonatal supporting cells and analysis by RNA-seq. (A) Sections through postnatal day 1 and 6 *LFng-GFP* transgenic mice. At both ages, the GFP transgene is expressed in border cells, inner phalangeal cells, outer pillar cells and all three rows of Deiters’ cells, but not inner pillar cells (arrowheads). Sections are counterstained with antibodies to MYOSIN VI to show hair cells and SOX2, PROX1, GLAST or NGFR to show distinct types of supporting cells (magenta). Scale bar 20 μm. (B) Representative FACS profile for sorting of P1 *LFng-GFP* transgenic cochleas. GFP intensity is shown on the x-axis, with four fractions (F1-F4) identified on the sorting profile. (C) QPCR analysis of the different fractions for expression of a hair cell marker (*Myo6*) and a supporting cell marker (*Prox1*). Cells falling in F3, which contained the highest *Prox1* signal and lowest *Myo6* signal were used in subsequent experiments. (D) Identification of transcripts enriched in the GFP^+^ fractions at P1 (left) and P6 (center) using the intersection of differential gene expression between GFP^+^ and GFP^-^ cells analyzed with DESeq and Cufflinks. 586 consensus GFP^+^ transcripts were identified at P1 and 508 at P6. The right Venn diagram shows the intersection of enriched GFP+ transcripts differentially expressed between P1 and P6 analyzed with DESeq and Cufflinks. 79 transcripts were enriched in P1 GFP^+^ cells compared to P6 GFP^+^ cells, whereas 259 transcripts were enriched in P6 GFP^+^ cells compared to P1 GFP^+^ cells. *p* < 0.01, q < 0.01 and log2(fold change) > 2.

Despite widespread interest in stimulating supporting cells to trans-differentiate into hair cells, relatively little is known about the genes expressed in supporting cells as they differentiate and mature. To identify supporting cell-specific transcripts in the neonatal mouse cochlea, we used *Lfng-GFP* BAC transgenic mice (Fig 1A) from the GENSAT project [28–30] to purify supporting cells by fluorescence-activated cell sorting. These mice show specific and strong GFP expression in most of the supporting cell types in the P1 and P6 cochlea, labeling border cells, inner phalangeal cells, outer pillar cells and all three rows of Deiters’ cells (Fig 1A). FACS analysis of the dissociated cell population revealed different populations with varying degrees of GFP fluorescence (Fig 1B). We collected only the brightest GFP^+^ cells for further analysis (Fig 1B “F3” fraction). Some cells expressed detectable but low levels of GFP fluorescence (Fig 1B; F1, F2 and F4 fractions) but were not collected for analysis, as they are likely to be inner pillar cells and inner hair cells that have been previously shown to express very low levels of this transgene [40]. We performed a preliminary validation of our collected fractions by examining expression of the hair cell transcript *Myo6* and the supporting cell transcript *Prox1* by Q-PCR (Fig 1C). The F3 fraction that was used for all subsequent analyses had the highest relative levels of *Prox1* expression and the lowest relative expression of *Myo6*. We obtained an average of 580±142 and 430±133 Lfng-EGFP^+^ cells per cochlea from P1 and P6 mice respectively. We prepared total RNA cDNA libraries from 50,000 cells per replicate from P1 and P6 mice and sequenced each library.

We compared the transcriptomes of GFP^+^ cells from P1 and P6 mice with those of the corresponding GFP^-^ populations. It is well-established that different methods to analyze RNA-seq data can give significantly different results. We therefore used two different analysis programs, Cufflinks [34] and DESeq [41] to normalize and compare differences in the mapped reads from our populations. We identified transcripts that were differentially expressed in GFP^+^ supporting cells compared to their GFP^-^ counterparts. Specifically, we found 2,964 significant differentially expressed genes (DEG) in the DESeq output and 2,318 DEG using Cufflinks for P1, and 2,180 DEG by DESeq and 1,542 DEG by Cufflinks for P6 (Fig 1D). We found a number of transcripts that were previously shown to be specifically expressed in supporting cells, such as *Lfng* itself, *Hes5*, *Fgfr3*, *Prox1*, *Sox2*, *Lgr5*, *Cdkn1b/p27*^*Kip1*^, and the *Notch1* receptor (S4 Table). We then focused on genes that were shown to be differentially expressed by both methods of analysis using similar criteria (p<0.01; FDR<0.01 and a fold change of at least 4). We identified 586 transcripts that were enriched in P1 GFP^+^ supporting cells compared to their GFP^-^ counterparts and 508 transcripts that were enriched in P6 GFP^+^ supporting cells compared to GFP^-^ cells (Fig 1D). We filtered these gene lists to generate candidates for further analysis by setting an arbitrary expression value of > 3000 RPKM. This yielded 277 supporting cell-enriched genes in the P1 cochlea and 202 at P6 (Tables 1 and 2 and S5 Table).

**Table 1:** Top 20 genes enriched in Lfng-GFP^+^ cells at P1.

Rank	Gene symbol	Mean GFP^+^ (RPKM)	Fold change (GFP^+^ vs GFP^-^)	*p-*adj
1	*Cysltr2*	4171.25	342.83	1.5E-135
2	*Hes5*	6663.27	306.73	1.68E-16
3	*Umodl1*	9261.97	227.61	2.68E-82
4	*Fgf3*	3272.03	219.78	5.68E-146
5	*Trhr*	5138.74	171.67	1.89E-236
6	*Gm5887*	7392.25	154.81	7.94E-254
7	*Pkd2l1*	8263.65	138.71	9.75E-255
8	*Fndc7*	5642.82	137.82	8.69E-171
9	*Pdzk1ip1*	6160.10	125.68	1.33E-37
10	*Slitrk6*	47604.15	120.46	3.23E-167
11	*Lfng*	109921.44	112.4	2.5E-252
12	*Agr3*	8355.38	104.39	6.16E-237
13	*Inhba*	4837.93	100.2	9.14E-208
14	*Slc6a14*	3419.69	95.49	6.99E-184
15	*Fgfr3*	86721.79	92.49	1.44E-275
16	*Nr4a3*	8069.48	91.92	1.34E-79
17	*Ntf3*	6712.00	78.59	9.04E-57
18	*Syt6*	16012.90	75.08	9.37E-239
19	*Slc27a2*	5244.69	70.04	1.46E-193
20	*Rab3b*	8196.90	67.84	7.35E-163

List of the 20 genes showing the greatest fold-change enrichment in P1 *Lfng-GFP*^*+*^ cells compared their GFP^-^ counterparts. The mean expression level (RPKM) is shown, together with the fold change versus GFP^-^ cells. *p-*adj = adjusted *p*-value for the difference between GFP^+^ and GFP^-^ populations.

**Table 2:** Top 20 genes enriched in Lfng-GFP^+^ cells at P6.

Rank	Gene symbol	Mean GFP^+^ (RPKM)	Fold change (GFP^+^ vs GFP^-^)	*p-*adj
1	*Umodl1*	87697.47	1022.11	1.86E-149
2	*Gm5887*	17389.04	850.84	1.06E-148
3	*Crhr1*	10718.38	310.72	1.17E-120
4	*Agr3*	4234.31	201.36	9.31E-24
5	*Tsga14*	79752.75	139.51	1.52E-104
6	*Wnt7a*	7143.40	125.77	1.26E-35
7	*Rassf6*	7298.55	77.97	2.34E-25
8	*Sapcd2*	15279.43	50.42	1.4E-13
9	*Dgkb*	5928.65	50.39	5.61E-69
10	*Slc6a14*	4914.60	45.72	1.62E-15
11	*Gdf6*	31099.52	45.01	2.29E-15
12	*Emx2*	3373.32	42.51	2.23E-19
13	*Tmprss3*	15912.06	42.17	1.45E-66
14	*Tppp*	4647.09	41.33	1.33E-62
15	*Otog*	126262.45	37.63	5.36E-67
16	*Slitrk6*	35908.27	36.59	1.69E-64
17	*Rnd1*	5759.25	34.17	4.57E-06
18	*Fgfr3*	49608.94	33.2	1.18E-18
19	*Prox1*	4088.87	32.69	4.72E-09
20	*Rorb*	4155.72	31.4	3.5E-55

List of the 20 genes showing the greatest fold-change enrichment in P6 *Lfng-GFP*^*+*^ cells compared their GFP^-^ counterparts. Ranking is based on the size of the fold change. The mean expression level (RPKM) is shown, together with the fold change versus GFP^-^ cells. *p-*adj = adjusted *p*-value for the difference between GFP^+^ and GFP^-^ populations

While 907 of the genes enriched in supporting cells were expressed at both P1 and P6, 976 were expressed only at P1 and 371 were expressed only at P6. Consistent with maturation of supporting cells prior to the onset of hearing, the expression of many supporting cell-specific genes changed during this developmental period. We identified differentially expressed supporting cell genes by comparing P1 and P6 Lfng-GFP^+^ samples using the same combined analysis with DESeq and Cufflinks described above. We identified 338 differentially expressed genes, of which 79 were enriched in P1 GFP^+^ supporting cells compared to their P6 counterparts and 259 genes that were enriched in P6 GFP^+^ supporting cells compared to P1 supporting cells (Fig 1D, Tables 3 and 4 and S6 Table).

**Table 3:** Top 20 differentially expressed genes enriched in P1 Lfng-GFP^+^ cells versus P6 Lfng-GFP^+^ cells.

Rank	Gene symbol	P1 Mean GFP^+^ (RPKM)	P6 Mean GFP^+^ (RPKM)	Fold change enrichment in P1 versus P6	*p*-adj
1	*Syt6*	10312.10	146.09	70.52	8.13E-87
2	*Cysltr2*	2686.38	81.44	32.90	2.99E-56
3	*Tmc5*	5343.56	223.78	23.92	3.58E-52
4	*Tal1*	2795.18	71.13	39.40	1.52E-48
5	*Pkd2l1*	5321.86	285.92	18.64	2.28E-47
6	*March4*	1390.15	45.89	30.27	1.81E-45
7	*Trhr*	3309.26	271.09	12.21	6.58E-36
8	*Angpt1*	4046.49	387.49	10.41	5.43E-32
9	*Chst15*	4208.43	392.24	10.70	6.02E-32
10	*Olfml3*	7644.27	532.96	14.32	2.36E-27
11	*Chrng*	1009.93	87.34	11.55	6.5E-27
12	*Raver2*	23804.79	3433.25	6.92	1.38E-25
13	*Fat3*	3525.47	418.60	8.40	2.26E-25
14	*Cdh4*	1950.64	231.45	8.46	1.14E-23
15	*Cxcl12*	6129.68	847.05	7.26	1.02E-22
16	*Sox11*	5314.63	764.77	6.96	1.41E-22
17	*St8sia2*	6179.44	890.31	6.96	1.87E-22
18	*Trh*	1840.37	261.91	7.01	1.02E-21
19	*Dpysl4*	9539.39	1636.75	5.82	2.46E-21
20	*Cyp26b1*	52399.13	9464.61	5.54	2.26E-20

List of the 20 genes showing the greatest fold-change enrichment in P1 *Lfng-GFP*^*+*^ cells compared P6 *Lfng-GFP*^*+*^ cells. Ranking is based on the *p* value. The mean expression level (RPKM) is shown, together with the fold change of enrichment in P1 versus P6 cells. *p-*adj = adjusted *p*-value for the difference between P1 and P6 populations.

**Table 4:** Top 20 differentially expressed genes enriched in P6 Lfng-GFP^+^ cells versus P1 Lfng-GFP^+^ cells.

Rank	Gene symbol	P1 Mean GFP^+^ (RPKM)	P6 Mean GFP^+^ (RPKM)	Fold change enrichment in P6 versus P1	*p*-adj
1	Kcnj16	639.37	14990.18	23.43	3.11E-56
2	Dgkb	188.78	4453.61	23.59	1.29E-52
3	Pcsk2	65.82	2669.74	40.50	3.66E-51
4	Grip2	47.68	1375.26	28.84	6.34E-48
5	Scd1	655.77	22369.52	34.06	1.07E-42
6	Crhr1	624.41	8053.18	12.91	4.54E-39
7	Crb2	109.85	2324.76	21.11	1.85E-38
8	Rasgrp1	288.59	3520.79	12.21	2.45E-34
9	Mmp28	98.40	1238.65	12.55	2.63E-30
10	Bhlhe40	171.20	2946.79	17.27	1.19E-29
11	Umodl1	5964.36	65915.69	11.08	2.59E-28
12	Pla2g4e	96.94	1113.05	11.47	2.88E-28
13	Stat5a	146.64	1498.22	10.20	3.54E-27
14	Abcc12	129.82	1283.89	9.92	3.23E-25
15	Aim1	237.75	1998.98	8.40	9.76E-25
16	Hr	966.69	6669.82	6.92	1.47E-24
17	Cntfr	85.05	1371.73	16.11	1.66E-24
18	Sorl1	2809.49	18130.06	6.45	3.25E-24
19	Col13a1	101.25	3235.28	32.00	3.36E-23
20	Klf9	287.49	2141.53	7.46	4.03E-23

List of the 20 genes showing the greatest fold-change enrichment in P6 *Lfng-GFP*^*+*^ cells compared P1 *Lfng-GFP*^*+*^ cells. Ranking is based on the *p* value. The mean expression level (RPKM) is shown, together with the fold change of enrichment in P6 versus P1 cells. *p-*adj = adjusted *p*-value for the difference between P6 and P1 populations.

We performed gene ontology (GO) and pathway analyses on our filtered list of P1 and P6 supporting cell genes using the DAVID bioinformatics suite [35, 37]. The biological processes terms most significantly represented in our lists of P1 genes were “neuron differentiation” (GO:0030182), “cell fate commitment” (GO:0045165) and “cell adhesion” (GO:0007155; Table 5). In P6 supporting cells, the most significant terms were “inner ear development” (GO:0048839), “regulation of cellular component biogenesis” (GO: 0044087) and “neuron differentiation” (GO:0030182; Table 6). To interpret the long list of biological process represented in our analysis, we summarized them using the REVIGO suite [39], showing only the most significantly represented GO terms (p<0.01; Tables 5 and 6). We found that GO terms including “Notch pathway” (GO:0007219) or its regulation (GO:0008593), “cell projection organization” (GO:00330030) and “cell component movement” (GO:0006928) were present in our P1 lists but were less represented in the P6 gene lists. In contrast, terms associated with cellular organization, such as “cellular component biogenesis” (GO:0044087), “regulation of protein polymerization” (GO:0032271), “negative regulation of proliferation” (GO:0008285) or “cytoskeleton organization” (GO:0007010) were more represented in P6 supporting cells, consistent with the morphological maturation of supporting cells at this time. Finally, DAVID analysis of biological processes terms represented in the list of genes that significantly changed in supporting cells between P1 and P6 showed that the most significantly represented terms were cell adhesion (GO:0007155), extracellular structure organization (GO:0043062) and hormone metabolic processes (GO:0042445). To summarize the list of biological process terms we used the REVIGO suite as described above (Table 7).

**Table 5:** Biological processes (BP) GO terms represented in the genes enriched in P1 Lfng-GFP^+^ cells.

Representative GO Term description (REVIGO Output)	Term ID	Included GO Term descriptions (DAVID Output)	Genes represented	log10 p value
**Neuron Differentiation**	GO:0030182	Neuron differentiation	*Enah*, *Fgfr3*, *Sox2*, *Uchl1*, *Rora*, *Jag1*, *Ephb1*, *Tgfb2*, *Nrcam*, *Efhd1*, *Tctn1*, *Sema3a*, *Ntf3*, *Ptprz1*, *Tgfbr1*, *Emx2*, *Ntng2*, *Rgnef*, *Notch3*, *Sall3*, *Notch1*, *Hes5*, *Ush1c*, *Id4*, *Slitrk6*, *Wnt7a*, *Igsf9*	-9.8358
	GO:0048839	Inner ear development	*Spry2*, *Fgfr3*, *Cdkn1b*, *Hes5*, *Sox2*, *Ush1c*, *Fgf10*, *Nr4a3*, *Jag1*, *Celsr1*, *Fzd6*, *Ptprq*	-7.2417
	GO:0048729	Tissue morphogenesis	*Enah*, *Fgfr3*, *Fgf10*, *Jag1*, *Celsr1*, *Nr4a3*, *Prox1*, *Fzd6*, *Tgfb2*, *Notch1*, *Sfrp1*, *Frem2*, *Lama5*, *Tctn1*, *Sema3a*, *Wnt7a*	-5.6775
	GO:0051094	Positive regulation of developmental process	*Tal1*, *Notch1*, *Fgfr3*, *Socs2*, *Ntf3*, *Sox2*, *Hey2*, *Jag1*, *Prox1*, *Igfbp3*, *Wnt7a*, *Tgfb2*	-3.5207
	GO:0001709	Cell fate determination	*Ntf3*, *Hes5*, *Gata3*, *Cyp26b1*, *Prox1*	-3.229
	GO:0035239	Tube morphogenesis	*Spry2*, *Notch1*, *Enah*, *Lama5*, *Fgf10*, *Cdh1*, *Tctn1*, *Nr4a3*, *Celsr1*, *Fzd6*	-3.045
	GO:0007605	Sensory perception of sound	*Spry2*, *Cdkn1b*, *Otof*, *Sox2*, *Otog*, *Ush1c*, *Tectb*	-2.8523
	GO:0022612	Gland morphogenesis	*Notch1*, *Sfrp1*, *Lama5*, *Fgf10*, *Cdh1*, *Sema3a*	-2.1185
	GO:0001763	Morphogenesis of a branching structure	*Spry2*, *Notch1*, *Sfrp1*, *Lama5*, *Fgf10*, *Spint1*, *Sema3a*	-2.0023
**Cell Adhesion**	GO:0007155	Cell adhesion	*Cldn7*, *Cldn9*, *Cldn6*, *Ninj1*, *Lmo7*, *Nedd9*, *Bcam*, *Cdh1*, *Tgfb2*, *Nrcam*, *Cd9*, *Fat3*, *Pvrl3*, *Ttyh1*, *Thbs1*, *F11r*, *Egfl6*, *Fblim1*, *Celsr2*, *Ptpru*, *Celsr1*, *Pgm5*, *Itga6*, *Hes5*, *Frem2*, *Lama5*, *Lsamp*, *Pkp3*, *Otog*, *Cntn1*, *Lamc2*	-9.2086
	GO:0016337	Cell-cell adhesion	*Cldn7*, *Cldn9*, *Cldn6*, *Lmo7*, *Celsr2*, *Cdh1*, *Celsr1*, *Ptpru*, *Tgfb2*, *Nrcam*, *Itga6*, *Fat3*, *Frem2*, *Pvrl3*, *Ttyh1*	-5.0319
**Cell Projection Organization**	GO:0030030	Cell projection organization	*Enah*, *Ntf3*, *Ptprz1*, *Uchl1*, *Ntng2*, *Rgnef*, *Ephb1*, *Tgfb2*, *Nrcam*, *Efhd1*, *Notch1*, *Itga6*, *Lama5*, *Ttyh1*, *Tctn1*, *Sema3a*, *Slitrk6*, *Igsf9*	-5.3666
	GO:0044087	Regulation of cellular component biogenesis	*Fmn1*, *Cdkn1b*, *Tgfbr1*, *Spnb1*, *Prox1*, *Wnt7a*, *Vill*	-2.723
**Notch Signaling Pathway**	GO:0007219	Notch signaling pathway	*Notch3*, *Notch1*, *Hey1*, *Heyl*, *Hey2*, *Cntn1*, *Jag1*	-3.8925
	GO:0008593	Regulation of Notch signaling pathway	*Sox2*, *Hey2*, *Fgf10*, *Jag1*	-3.7913
	GO:0016055	Wnt receptor signaling pathway	*Fzd9*, *Sfrp1*, *Ccdc88c*, *Kremen1*, *Celsr2*, *Frzb*, *Fzd4*, *Wnt7a*, *Fzd6*	-3.2014
	GO:0007167	Enzyme linked receptor protein signaling pathway	*Ephb6*, *Fgfr3*, *Erbb4*, *Id1*, *Myo1e*, *Tgfbr1*, *Fgf10*, *Angpt1*, *Ephb1*, *Fgf3*, *Tgfb2*	-2.1651
**Cell Motion**	GO:0006928	Cell motion	*Enah*, *Ntf3*, *Tgfbr1*, *Emx2*, *Fgf10*, *Ephb1*, *Tgfb2*, *Nrcam*, *Cd9*, *Syne2*, *Itga6*, *Lama5*, *St14*, *Sema3a*	-2.5456

Summary of biological processes GO terms that were significantly represented (p<0.01) in 277 genes enriched in P1 *Lfng-EGFP*^+^ cells (filtered lists: present in DESeq and Cufflinks outputs, RPKM>3000 and FC>4) relative to their GFP^-^ counterparts. The biological processes represented were obtained with the DAVID bioinformatics suite and then summarized with the REVIGO tool.

**Table 6:** Biological processes (BP) GO terms represented in the genes enriched in P6 Lfng-GFP^+^ cells.

Representative GO Term description (REVIGO Output)	Term ID	Included GO Term descriptions (DAVID Output)	Genes represented	log10 p value
**Inner Ear Development**	GO:0048839	Inner ear development	*Spry2*, *Fgfr3*, *Cdkn1b*, *Sox2*, *Ush1c*, *Fgf10*, *Nr4a3*, *Jag1*, *Gjb6*	-5.363
	GO:0030182	Neuron differentiation	*Fgfr3*, *Ptprz1*, *Sox2*, *Uchl1*, *Emx2*, *Rorb*, *Jag1*, *Rora*, *Sall3*, *Nrcam*, *Efhd1*, *Ush1c*, *Id4*, *Stmn1*, *Slitrk6*, *Igsf9*, *Wnt7a*	-5.2594
	GO:0007605	Sensory perception of sound	*Spry2*, *Cdkn1b*, *Otof*, *Sox2*, *Otog*, *Ush1c*, *Gjb6*, *Tectb*	-4.5074
	GO:0051094	Positive regulation of developmental process	*Wnt7b*, *Fgfr3*, *Tnfrsf12a*, *Sox2*, *Hey2*, *Jag1*, *Prox1*, *Igfbp3*, *Vash2*, *Wnt7a*	-3.3182
	GO:0051130	Positive regulation of cellular component organization	*Cdkn1b*, *Tnfrsf12a*, *Tppp*, *Fgf10*, *Prox1*, *Wnt7a*	-2.0054
**Regulation of Cellular Component Biogenesis**	GO:0044087	Regulation of cellular component biogenesis	*Cdkn1b*, *Gsn*, *Tppp*, *Capg*, *Stmn1*, *Prox1*, *Wnt7a*, *Epb4*.*9*, *Vill*	-5.326
	GO:0032271	Regulation of protein polymerization	*Cdkn1b*, *Gsn*, *Tppp*, *Capg*, *Stmn1*, *Epb4*.*9*, *Vill*	-4.5105
	GO:0008593	Regulation of Notch signaling pathway	*Sox2*, *Hey2*, *Fgf10*, *Jag1*	-4.1932
	GO:0051493	Regulation of cytoskeleton organization	*Cdkn1b*, *Gsn*, *Capg*, *Stmn1*, *Mid1ip1*, *Prox1*, *Epb4*.*9*, *Vill*	-4.0483
	GO:0051129	Negative regulation of cellular component organization	*Gsn*, *Snph*, *Capg*, *Stmn1*, *Mid1ip1*, *Epb4*.*9*, *Vill*	-3.3247
	GO:0043244	Regulation of protein complex disassembly	*Gsn*, *Capg*, *Mid1ip1*, *Epb4*.*9*, *Vill*	-2.9542
	GO:0033043	Regulation of organelle organization	*Cdkn1b*, *Gsn*, *Capg*, *Stmn1*, *Mid1ip1*, *Prox1*, *Epb4*.*9*, *Vill*	-2.8818
	GO:0008285	Negative regulation of cell proliferation	*Cd9*, *Spry2*, *Fgfr3*, *Cdkn1b*, *Gata3*, *Fgf10*, *Gjb6*, *Prox1*, *Igfbp3*	-2.5565
	GO:0032970	Regulation of actin filament-based process	*Gsn*, *Capg*, *Prox1*, *Epb4*.*9*, *Vill*	-2.3922
	GO:0007010	Cytoskeleton organization	*Shroom1*, *Gsn*, *Tppp*, *Epb4*.*1*, *Ush1c*, *Stmn1*, *Prox1*, *Epb4*.*9*, *Vill*, *Tmod1*	-2.0821
**Cell Adhesion**	GO:0007155	Cell adhesion	*F11r*, *Cldn7*, *Cldn9*, *Col13a1*, *Tnfrsf12a*, *Cldn3*, *Nedd9*, *Fblim1*, *Bcam*, *Nrcam*, *Cd9*, *Wnt7b*, *Pvrl3*, *Pkp3*, *Otog*, *Ttyh1*	-3.0164

Summary of biological processes GO terms that were significantly represented (p<0.01) in 202 genes enriched in P6 LfngGFP+ cells (filtered lists: present in DESeq and Cufflinks outputs, RPKM>3000 and FC>4) relative to their GFP- counterparts. The biological processes represented were obtained with the DAVID bioinformatics suite and then summarized with the REVIGO tool.

**Table 7:** Biological processes (BP) GO terms significantly represented in the list of DEG between P1 and P6 Lfng-GFP^+^ cells.

Representative GO Term description (REVIGO Output)	Term ID	Included GO Term descriptions (DAVID Output)	Genes represented	log10 p value
**Cell adhesion**	GO:0007155	Cell adhesion	*Ibsp*, *Col13a1*, *Tnc*, *Bcam*, *Vtn*, *Itgb3*, *Cdh3*, *Cdh4*, *Lama1*, *Wisp1*, *Fat3*, *Hes5*, *Itga8*, *Thbs1*, *Col8a1*	-4.1292
	GO:0030155	Regulation of cell adhesion	*Lama1*, *Stat5a*, *Vtn*, *Thbs1*, *Col8a1*	-2.264
**Extracellular structure organization**	GO:0043062	Extracellular structure organization	*Ibsp*, *Drp2*, *Itga8*, *Tnc*, *Eln*, *P2rx2*, *Vtn*	-3.0499
**Hormone metabolic process**	GO:0042445	Hormone metabolic process	*Aldh1a1*, *Pcsk2*, *Dio2*, *Chst8*, *Cyp26b1*	-2.3309

Summary of biological processes GO terms that were significantly represented (p<0.01) in 338 differentially expressed genes in P1 relative to P6 LfngGFP+ cells (present in DESeq and Cufflinks outputs, enriched in P1 or P6 and FC>4). The biological processes represented were obtained with the DAVID bioinformatics suite and then summarized with the REVIGO tool.

To further understand the genes responsible for supporting cell maturation, we compared our P1 and P6 transcriptomes, focusing on genes related to cell fate commitment, cellular component biogenesis and cytoskeletal organization. As a first step in assembling a supporting cell gene regulatory network, we found 36 transcription factors expressed significantly in P1 supporting cells, of which only nine (*Sox2*, *Sox6*, *Prox1*, *Tal1*, *Isl2*, *Atoh1*, *Hes5*, *Sall1*, and *Gata3*) were related to cell fate commitment on the basis of gene ontology. At P6 we found 32 enriched transcription factors, with only five related to cell fate commitment (*Hes5*, *Sall1*, *Gata3*, *Sox2* and *Prox1*). Comparing both ages showed seven transcription factors (*Tal1*, *Atoh1*, *Hes5*, *Egr4*, *Sox11*, *Gm98*, and *Hmga2*) changing significantly from P1 to P6.

We also looked for known downstream genes that might be associated with supporting cell maturation. Potential candidates enriched in P6 supporting cells included microtubule associated genes (*Tsga14*, *Stmn1*, *Shroom1* and *Tppp*) [42–45] actin microfilament associated genes (*Shroom1*, *Capg and Gsn*) [44, 46], and *Tnfrsf12a*, a gene implicated in extracellular matrix adhesion [47]. Of these genes, only *Shroom1* and *Tppp* were also enriched in P1 supporting cells. Finally, we found 20 known deafness genes enriched in supporting cells at P1 (*Tmprss3*, *Cabp2*, *Ush1c*, *Ildr1*, *Otog*, *Smp*, *Pou4f3*, *Gipc3*, *Grxcr2*, *Grhl2*, *Otof*, *Loxhd1*, *Strc*, *Grxcr1*, *Myo3a*, *Ptprq*, *Myh14*, *Marveld2*, *Pcdh15* and *Fam65b*), 18 enriched at P6 (*Tmprss3*, *Smpx*, *Otog*, *P2rx2*, *Ildr1*, *Strc*, *Loxhd1*, *Ush1c*, *Pou4f3*, *Slc26a5*, *Grxcr2*, *Pcdh15*, *Grhl2*, *Otof*, *Ceacam16*, *Tmc1*, *Marveld2* and *Gjb6*) and just five changing significantly between P1 to P6 (*Ceacam16*, *Tnc*, *Otoa*, *Slc26a5* and *P2rx2*).

**Fig 2:**
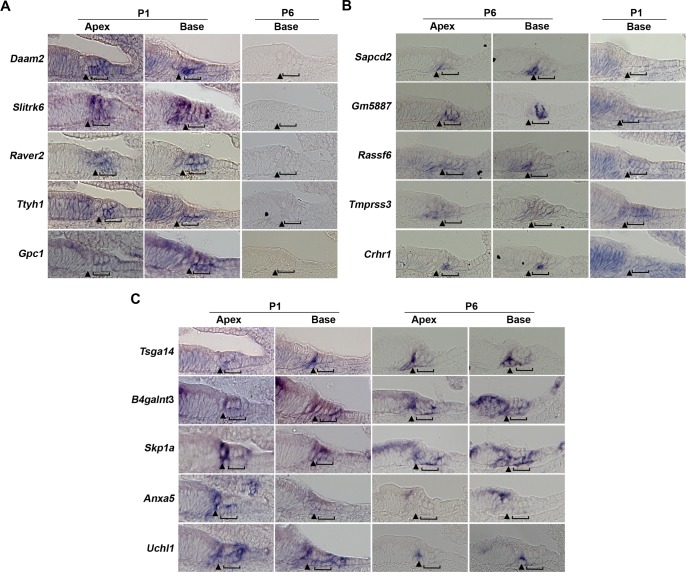
In situ validation of supporting cell-specific transcripts. Examples of supporting cell genes enriched in **(A)** P1 supporting cells with basal P6 sections to show negative expression **(B)** P6 supporting cells with basal P1 sections to show negative expression **(C)** Both P1 and P6 supporting cells, with sections to show both apical (less mature) and basal (more mature) regions at each age. Brackets: Deiters’ cells; Arrowhead: pillar cell region; horizontal line, greater epithelial ridge region.

To further validate the expression of our supporting cell-specific genes, we examined the expression of selected genes on cochlear sections of P1 and P6 mice by in situ hybridization. We chose genes on the basis of their RPKM values and significant enrichment at either age (Fig 2; S7 Table). We previously used in situ hybridization to validate hair cell-specific transcripts, and found that only about 50% of hair cell-enriched transcripts gave detectable, hair cell-specific expression patterns [48]. Similarly, in our supporting cell analysis, we found many genes enriched in supporting cells on the basis of RNA-seq that gave broader patterns of expression in other cochlear cell types (S7 Table). We evaluated five genes that were specifically expressed at P1 (*Daam2*, *Raver2*, *Slitrk6*, *Ttyh1* and *Gpc1*), five genes specifically expressed at P6 (*Sapcd2*, *Gm5887*, *Rassf6*, *Tmprss3* and *Crhr1*) and five genes expressed at both ages (*Tsga14*, *B4galnt3*, *Skp1a*, *Anxa5* and *Uchl1*). Of those only *Slitrk6* expression has been previously characterized in the cochlea [49]. In situ analysis revealed a wide variety of expression patterns, with some genes expressed broadly in most supporting cell types (*B4galnt3*, *Skp1a*) at both ages, others restricted to one supporting cell subtype at both ages (e.g. *Tsga14* in pillar cells), whereas other genes were broadly expressed at only one age (*Slitrk6* at P1) or restricted to a particular sub-population at only one age (*Gm5887* in Deiters’ cells at P6). We also observed apical-basal gradients of expression for some genes at both ages (Fig 2). In summary, in addition to validating our RNA-seq data, our in situ hybridization revealed the presence of spatially and temporally varying and sub-population-specific gene expression for many of our supporting cell transcripts.

### Mouse cochlear supporting cells become almost completely unresponsive to blockade of Notch signaling by six days of age

The Notch signaling pathway plays a critical role in establishing hair cell and supporting cell identity during the differentiation of inner ear sensory tissue [50]. Specifically, hair cells expressing the DLL1, DLL3 and JAG2 ligands deliver a Notch signal to neighboring supporting cells that prevents their differentiation into hair cells. Accordingly, genetic inactivation of Notch receptors, their ligands or downstream transcriptional effectors in the inner ear causes an over-production of hair cells at the expense of supporting cells [51–56]. Moreover, blockade of the Notch signaling pathway through pharmacological inhibition of the gamma secretase complex required for Notch receptor cleavage or antibody blockade of Notch receptors themselves can also cause supporting cells to trans-differentiate into hair cells [13, 17–20]. However, the ability of Notch inhibition to cause supporting cell trans-differentiation decline rapidly with age, and can even be observed in supporting cells of different maturational states along the basal-apical axis of the neonatal cochlea [19]. We have previously shown that the expression of some components of the Notch pathway is reduced in mouse cochlear supporting cells in the first postnatal week [19]. However, it is formally possible that significant numbers of transcriptional changes still occur when the Notch pathway is blocked in P6 supporting cells, but these are nevertheless insufficient to drive trans-differentiation to hair cells.

We first assessed the expression of known Notch pathway genes in our P1 and P6 data sets. We found many Notch pathway genes enriched in supporting cells, and many of these were down-regulated between P1 and P6 as previously described [19] (Table 8). To test whether changes in Notch responsiveness of supporting cells between birth and P6 are associated with changes in the transcriptional response to Notch blockade, we cultured cochleas from newborn (P0) or P5 mice in the presence or absence of the gamma secretase inhibitor DAPT for 24 hours as previously described [19]. Our previous work showed that DAPT, which prevents gamma secretase-mediated cleavage of many proteins in addition to Notch receptors, has the same effect on neonatal cochlear cultures as Notch1 blocking antibodies [19]. At the end of the culture period, we dissociated the cultured cochleas and purified GFP^+^ and GFP^-^ cells by fluorescence-activated cell sorting. We reasoned that culturing P0 and P5 cochleas for 24 hours was the most appropriate way to compare their transcriptomes to the acutely isolated P1 and P6 supporting cells described above. The GFP label was stable and retained by supporting cells during this 24 hour period of Notch blockade, allowing us to isolate supporting cells as they began to trans-differentiate into hair cells (S1A–S1C Fig). We isolated between 500–600 supporting cells per cochlea in each condition (DMSO or DAPT) at P1, and between 300–400 supporting cells per cochlea at P6. Between 10,000–20,000 purified supporting cells were used to create RNA-seq libraries at each age and condition, with duplicate samples being analyzed. GFP^+^ and GFP^-^ cell populations from P0 and P5 supporting cells cultured in DAPT or DMSO vehicle were analyzed by RNA-seq. Mapped and normalized data sets were compared to identify supporting cell transcripts that were significantly up- or down-regulated in P0 supporting cells that were treated with DAPT compared to DMSO, and a similar analysis was performed for our P5 samples.

**Table 8:** Notch and Wnt pathway genes expressed in Lfng-GFP^+^ cells at P1 and P6.

	*Postnatal Day 1*			*Postnatal Day 6*		
Genes	Expression in Lfng-GFP^+^ (RPKM)	Fold change (GFP^+^vs GFP^-^)	*p-*adj	Expression in Lfng-GFP^+^ (RPKM)	Fold change (GFP^+^vs GFP^-^)	*p-*adj
*Lfng*	109921.44	112.40	2.5E-252	117587	81.79	2.31E-10
*Hey1*	22093.29	25.21	6.75E-160	9892	4.35	0.000014
*Jag1*	81967.23	19.58	2.12E-105	47865	17.31	9.94E-43
*Hey2*	7358.57	12.92	1.09E-102	6735	10.27	1.79E-24
*Mycl1*	7233.13	10.13	1.11E-86	6790	7.76	4.09E-08
*Notch1*	47252.67	4.36	1E-44	14809.73	1.650383	0.208748
*Notch3*	21210.78	4.03	2.78E-38	8181.368	2.125937	0.000264
*Cntn1*	9585.18	5.58	1.92E-34	4419	4.25	1.78E-12
*Mfng*	1254.47	6.30	9.05E-17	731	5.14	9.28E-08
*Hes5*	6663.27	306.73	1.68E-16	737	174.97	4.39E-24
*Hr*	1500.99	4.81	1.34E-11	8878.668	3.452246	2.05E-09
*Lor*	364.97	8.87	9.93E-08	45.26282	1.175772	1
*Wnt7a*	10897.69	46.84	1.3E-190	7143.40	125.77	1.26E-35
*Fzd4*	10204.53	35.42	7.98E-66	3761.40	5.64	2.77E-16
*Frzb*	13318.59	18.55	1.41E-134	2707.837	0.901449	1
*Kremen1*	55934.39	16.31	1.17E-134	50543.90	5.94	2.14E-18
*Sfrp1*	53947.70	9.40	1.6E-87	17549.5299	6.98	3.18E-22
*Fzd6*	6100.32	4.52	2.92E-41	7130.342	3.684441	4.31E-10
*Wnt7b*	2444.61	4.26	1.43E-11	3047.78699	7.61	1.5E-13

Known members of the Notch and Wnt signaling pathways expressed in either P1 or P6 *LFng-GFP*^*+*^ cells. The mean expression level (RPKM) is shown, together with the fold change versus GFP^-^ cells. *p-*adj = adjusted *p*-value for the difference between GFP^+^ and GFP^-^ populations at each age.

**Fig 3:**
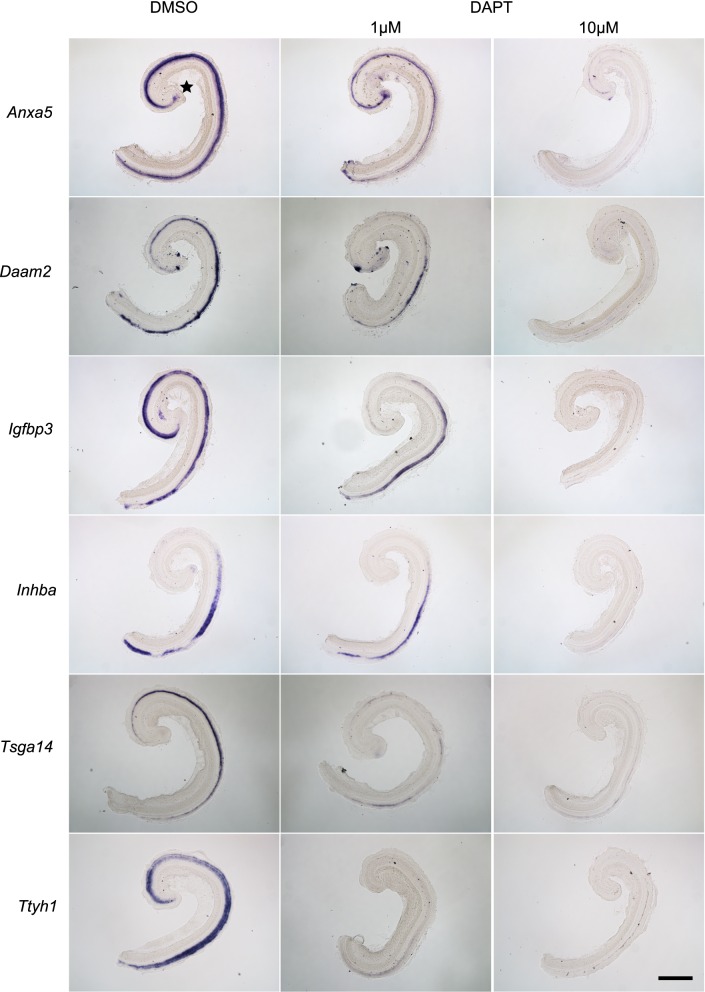
Gene expression changes in P0 supporting cells in response to Notch inhibition. Demonstration of rapid down-regulation by *in* situ hybridization of six supporting cell-specific genes in P0 cochleas after 24 hours of culture in DAPT versus DMSO controls. Scale Bar: 500 μm. Star: apex tip.

We used DESeq to identify 2,088 supporting cell-specific genes that were significantly changed after DAPT treatment of P0 cochleas (S1D Fig), with 1032 transcripts being down-regulated (Table 9) and 1056 transcripts up-regulated (Table 10 and S8 Table). In agreement with the observed trans-differentiation of neonatal supporting cells after Notch blockade, 237 of the transcripts up-regulated by DAPT were among the 304 genes we had previously shown to be strongly enriched in hair cells [48] and having RPKM higher than 3000. Moreover, consistent with our previous observation that DAPT treatment of cochlear cultures largely works through the Notch pathway [19], GO analysis of the down-regulated supporting cell-specific genes after DAPT treatment featured many Notch pathway members (Table 11). In stark contrast, when P5 cochleas were treated with DAPT, we only observed 20 supporting cell-specific genes that were changed significantly, with 2 transcripts up-regulated and 18 down-regulated (S1D Fig and Table 12). Moreover, many of these genes were expressed at extremely low levels compared to younger supporting cells–for example, although *Hes5* still showed a significant down-regulation after DAPT treatment of P5 cochleas, its normalized expression (in RPKM) changed from 120 to 3 after DAPT treatment, whereas its levels in P1 supporting cells was over 6,600 (Table 1). To validate our data sets, we cultured P0 cochleas in the presence or absence of DAPT for 24 hours and then analyzed changes in some of the supporting cell-specific genes by whole mount *in situ* hybridization (Fig 3). Of the genes evaluated, *Ttyh1* exhibited the strongest change in *in situ* signal, consistent with the RNA-seq data where its normalized level in RPKM dropped from 13941 to 1689. Similarly *Anxa5*, *Daam2*, *Inhba*, *Igfbp3 and Tsga14* also decreased and resembled their decrease in RPKM seen after DAPT treatment. Consistent with our previous observations that the apex of the neonatal cochlea is more sensitive to Notch inhibition than the base [19], the genes evaluated showed more significant decreases in signal in the apex of the DAPT-treated cochleas than the base with the exception of *Anxa5* that showed a more diffuse decrease in the signal from apex to base. Taken together, our data confirm that the Notch signaling pathway is functionally dismantled in supporting cells between P1 and P6 such that Notch inhibition leads to barely perceptible transcriptional changes in P6 mature supporting cells.

**Table 9:** Top 20 down-regulated genes after DAPT treatment in P0 Lfng-GFP^+^ cells.

Rank	Gene symbol	DMSO Mean GFP^+^ (RPKM)	DAPT Mean GFP^+^ (RPKM)	Fold change DAPT vs DMSO	*p*-adj
**1**	*Tal1*	2983.37	2.56	0.0009	8.77E-181
**2**	*Cysltr2*	3834.07	22.16	0.0058	9.07E-148
**3**	*Pdzk1ip1*	2814.84	19.02	0.0068	5.70E-127
**4**	*Heyl*	4828.46	224.34	0.0464	1.55E-102
**5**	*Prss23*	4249.27	215.01	0.0508	1.72E-95
**6**	*Gstm1*	4572.23	331.06	0.0723	2.42E-80
**7**	*Gm10800*	74333.61	8438.92	0.1134	2.45E-77
**8**	*2310022B05Rik*	8186.49	813.73	0.0994	3.14E-74
**9**	*Dgat2*	1061.61	21.63	0.0203	5.69E-70
**10**	*Syt6*	6254.68	431.84	0.0689	6.15E-70
**11**	*Slitrk2*	1253.99	38.20	0.0304	3.02E-69
**12**	*Ttyh1*	13940.83	1688.84	0.1207	8.12E-68
**13**	*Pdgfrb*	2128.79	118.91	0.0559	2.74E-67
**14**	*Ccdc80*	3066.68	252.98	0.0825	2.10E-66
**15**	*AC131780*.*2*	6376.16	723.02	0.1134	2.31E-64
**16**	*Rab3b*	5451.58	600.43	0.1103	8.92E-64
**17**	*Nrarp*	1389.90	68.84	0.0494	2.34E-61
**18**	*Dkk3*	3824.89	424.10	0.1111	2.32E-58
**19**	*Sema5a*	2652.79	252.04	0.0947	6.94E-56
**20**	*Eno1*	3363.46	382.22	0.1134	3.34E-55

List of the 20 genes showing the greatest down-regulation in P0 *Lfng-GFP*^*+*^ cells after 24 hours DAPT treatment compared P0 *Lfng-GFP*^*+*^ cells treated with DMSO vehicle. Ranking is based on the *p* value. The mean expression level (RPKM) is shown, together with the fold change versus DMSO-treated cells. *p-*adj = adjusted *p*-value for the difference between DAPT and DMSO-treated populations.

**Table 10:** Top 20 up-regulated genes after DAPT treatment in P0 Lfng-GFP^+^ cells.

Rank	Gene symbol	DMSO Mean GFP^+^ (RPKM)	DAPT Mean GFP^+^ (RPKM)	Fold change DAPT vs DMSO	*p*-adj
**1**	*AC123795*.*1*	134.87	18009.79	133.44	3.21E-186
**2**	*Scn11a*	83.16	6687.08	80.45	8.07E-178
**3**	*Ush2a*	685.76	26222.34	38.32	1.77E-168
**4**	*Fam70b*	207.23	7185.86	34.78	9.23E-138
**5**	*Vwa5b2*	101.49	4564.69	44.94	7.78E-136
**6**	*Srrm4*	249.11	6795.38	27.28	9.01E-124
**7**	*P2rx3*	49.28	2547.23	51.63	4.06E-115
**8**	*Gas6*	251.21	5474.75	21.86	3.31E-97
**9**	*Myo3a*	365.31	6215.50	17.03	1.57E-96
**10**	*Thsd7b*	457.96	11936.04	25.99	5.86E-95
**11**	*Eya2*	33.79	1702.55	50.56	5.03E-94
**12**	*6330503K22Rik*	1044.82	19337.03	18.51	4.62E-88
**13**	*Gadd45g*	31.74	1444.82	45.57	6.97E-84
**14**	*Mobkl2b*	307.36	4600.97	14.93	8.34E-84
**15**	*Myo7a*	348.85	5023.23	14.42	2.21E-83
**16**	*Atoh1*	133.28	5021.72	37.79	2.49E-79
**17**	*Atp8a1*	2348.26	22393.66	9.51	7.43E-79
**18**	*Pole*	1944.59	18385.23	9.45	3.89E-77
**19**	*Jag2*	98.59	3275.67	33.13	1.01E-75
**20**	*Slc16a5*	58.11	1531.35	26.35	1.14E-71

List of the 20 genes showing the greatest up-regulation in P0 *Lfng-GFP*^*+*^ cells after 24 hours DAPT treatment compared P0 *Lfng-GFP*^*+*^ cells treated with DMSO vehicle. Ranking is based on the *p* value. The mean expression level (RPKM) is shown, together with the fold change versus DMSO-treated cells. *p-*adj = adjusted *p*-value for the difference between DAPT and DMSO-treated populations.

**Table 11:** Biological processes (BP) GO terms represented in the list of transcripts down-regulated after DAPT treatment of P0 Lfng-GFP^+^cells that were also enriched in P1 Lfng-GFP^+^cells.

Representative GO Term description (REVIGO Output)	Term ID	Included GO Term descriptions (DAVID Output)	Genes represented	log10 p value
Notch signaling pathway	GO:0007219	Notch signaling pathway	*Notch3*, *Notch1*, *Hey1*, *Heyl*, *Hey2*, *Cntn1*, *Jag1*	-6.4222
	GO:0008593	Regulation of Notch signaling pathway	*Hey2*, *Fgf10*, *Jag1*	-3.1195
	GO:0016055	Wnt signaling pathway	*Fzd9*, *Sfrp1*, *Frzb*, *Fzd4*, *Wnt7a*	-2.3026
Cell fate commitment	GO:0045165	Cell fate commitment	*Notch3*, *Tal1*, *Notch1*, *Erbb4*, *Hes5*, *Fgf10*, *Sox6*, *Tgfb2*	-4.91
	GO:0048729	Tissue morphogenesis	*Notch1*, *Sfrp1*, *Fgf10*, *Sema3a*, *Nr4a3*, *Jag1*, *Wnt7a*, *Tgfb2*	-3.5865
	GO:0051094	Positive regulation of developmental process	*Tal1*, *Notch1*, *Hey2*, *Jag1*, *Igfbp3*, *Wnt7a*, *Tgfb2*	-3.0288
Cell adhesion	GO:0007155	Cell adhesion	*Egfl6*, *Fblim1*, *Tgfb2*, *Cd9*, *Pgm5*, *Itga6*, *Hes5*, *Fat3*, *Lsamp*, *Ttyh1*, *Cntn1*, *Lamc2*, *Thbs1*	-4.4589
Cell projection organization	GO:0030030	Cell projection organization	*Notch1*, *Itga6*, *Ptprz1*, *Ttyh1*, *Sema3a*, *Slitrk6*, *Tgfb2*	-2.1669

Summary of biological processes GO terms that were significantly represented (p<0.01) in 97 genes enriched in P1 LfngGFP+ cells (filtered list: present in both DESeq and Cufflinks outputs, RPKM>3000, FC>4) and down regulated in P0 LfngGFP+ DAPT 24 hours treated cells (present in DESeq or Cufflinks outputs and FC>2) relative to their DMSO treated counterparts. The biological processes represented were obtained with the DAVID bioinformatics suite and then summarized with the REVIGO tool.

**Table 12:** All significantly up- or down-regulated genes after DAPT treatment in P5 Lfng-GFP^+^ cells.

Rank	Gene symbol	DMSO Mean GFP^+^ (RPKM)	DAPT Mean GFP^+^ (RPKM)	Fold change DAPT vs DMSO	*p*-adj
1	*Fabp7 †*	2378.03	558.00	0.23	3.89E-10
2	*Ttyh1*	5568.39	1810.79	0.33	1.06E-07
3	*2310022B05Rik*	3060.16	1069.78	0.35	1.43E-06
4	*Hes5 †*	120.67	3.21	0.03	1.43E-06
5	*Fzd9*	698.27	171.68	0.25	1.89E-06
6	*Ppp1r2*	8051.64	3088.33	0.38	5.43E-06
7	*Slc6a14*	1948.23	716.35	0.37	5.62E-05
8	*Cysltr2*	157.51	11.46	0.07	0.00011759
9	*Efr3b*	866.96	302.29	0.35	0.0002018
10	*Sez6l*	523.69	148.11	0.28	0.0002018
11	*Ednrb*	1686.11	622.61	0.37	0.0002018
12	*Daam2*	1579.09	376.46	0.24	0.00037462
13	*Eno1*	2183.19	929.87	0.43	0.00057668
14	*Slc2a9*	346.76	53.92	0.15	0.00122798
15	*Pak6 †*	141.12	16.16	0.11	0.00431559
16	*RP23-218C2*.*3*	75.13	3.41	0.05	0.00637716
17	*Srebf1 †*	3809.42	1780.78	0.47	0.00637716
18	*2610020H08Rik †*	3075.63	1446.24	0.47	0.0068551
19	*Dpysl2*	1661.82	3693.20	2.22	0.00120881
20	*Irf4 †*	21.31	188.19	8.82	0.0050224

List of all genes showing the significant up- or down-regulation in P5 *Lfng-GFP*^*+*^ cells after 24 hours DAPT treatment compared P5 *Lfng-GFP*^*+*^ cells treated with DMSO vehicle. Ranking is based on the *p* value. The mean expression level (RPKM) is shown, together with the fold change versus DMSO-treated cells. *p-*adj = adjusted *p*-value for the difference between DAPT and DMSO-treated populations.

† = genes also showing a significant change in DAPT-treated P0 cells (p<0.01).

## Discussion

Mammalian supporting cells undergo a remarkable morphological transformation before the onset of hearing that includes the formation of phalangeal processes by Deiters’ cells and inner phalangeal cells, the integration of supporting cell apical processes into the reticular lamina and the formation of the tunnel of Corti by pillar cells [27, 57, 58]. Although these morphological changes have been well-characterized, the molecular basis of this maturation is far less clear. While much recent attention has been paid to understanding the transcriptome of cochlear and vestibular hair cells, far less is known about the genes expressed by supporting cells as they differentiate and mature. Since the loss of regenerative potential of mouse supporting cells occurs over a similar time period as their maturation [1, 58], it is likely that identifying maturational changes in the supporting cell transcriptome may provide insights into the failure of mammalian hair cell regeneration.

We have characterized the transcriptome of mouse cochlear supporting cells in the first postnatal week. Using *LFng-GFP* transgenic mice, we were able to isolate all major supporting cell populations from P1 and P6 mice with the exception of inner pillar cells (Fig 1A). Using stringent criteria, we identified approximately 500 genes that were significantly enriched in P1 and P6 supporting cells. Although the gene expression profiles differed significantly between P1 to P6, there were many genes expressed in supporting cells at both stages. Instead of a radically different gene expression profile that might be expected between different cell types, the changes observed from P1 to P6 with GO analysis instead showed maturational changes of genes involved in regulation of the cell cycle, differentiation, the cytoskeleton and extracellular matrix. We identified a number of factors associated with maturation that has not been previously identified in the ear, such as *Tsga14*, *Stmn1*, *Tppp*, *Shroom1*, *Capg*, *Gsn* and *Tnfrsf12a*. Of note, one of the transcripts most strongly enriched in supporting cells at both P1 and P6 was the *Fgfr3* FGF receptor. FGFR3 signaling in pillar and Deiters’ cells is important for both the induction and maintenance of the identity of these supporting cells [59–62]. Strikingly, this signaling continues to be required for supporting cell identity in postnatal life, as mutations that change FGFR3 ligand specificity can cause Deiters’ cell–pillar cell transformations after the onset of hearing [63].

Our in situ analysis revealed a large amount of heterogeneity in expression patterns between different supporting cell genes. Although some genes were expressed in many supporting cell types, others were confined to just a few cell types such as pillar cells. In particular, many of the genes enriched in P6 supporting cells were localized to either pillar cells (*Sapcd2*, *Crhr1*, *Rassf6*) or Deiters’ cells (*Gm5887*). Recent advances in single cell transcriptome analysis have started to provide insights into supporting cell sub-populations [40, 64], and the availability of new markers for individual supporting cell types will allow for a more detailed characterization of their transcriptomes. Moreover, the known variations in hair cell gene expression along the tonotopic axis of the cochlea [65–68] are likely to have counterparts in the surrounding supporting cells, and it is likely that single cell analysis of supporting cell transcriptomes may reveal such gradients in the future.

Our study also confirms the importance of expression-based validation of RNA-seq data. In a previous study, we showed that only about 50% of genes shown to be enriched in neonatal hair cells gave specific or detectable signal in hair cells by in situ hybridization [48]. In the present study, we once again showed that only approximately 50% of genes identified as being enriched in supporting cells actually gave a specific in situ signal. While this may be due in part on technical artifacts arising from the choice of in situ probes or hybridization conditions, it is also a consequence of focusing on the degree of *differential* rather than *absolute* expression, whereby broadly expressed genes may still be expressed at higher levels in supporting cells.

Both the Notch and Wnt pathways have been suggested to regulate the differentiation of hair cells and supporting cells [50, 69] and manipulation of both pathways has been reported to increase both supporting cell proliferation and trans-differentiation into hair cells [13, 50, 52]. We previously showed that the mouse cochlear supporting cells become unresponsive to Notch inhibition in the first postnatal week, and that at least some components of the Notch pathway are down-regulated in supporting cells during this period [19]. Our current data confirms a clear down-regulation of signaling components in both the Notch and Wnt pathways between P1 and P6 (Table 8), which suggests that the number of transcriptional changes in supporting cells following Notch inhibition in P5 cochlear cultures was likely to be small. Our RNA-seq data of DAPT-treated cochleas now provide a striking confirmation of this hypothesis: although over 2,000 genes were either up- or down-regulated in supporting cells by DAPT treatment of P0 cochlear tissue, we only saw 20 genes change in treated P5 cochlear supporting cells. Moreover, of these 20 genes, only 13 had expression levels >500 RPKM. These results suggest that towards the end of the first postnatal week, blockade of the Notch pathway has essentially no effect on supporting cell transcription. However, we would caution that our experiments were performed on intact cochlear tissue in which hair cells remained alive, and it is possible that the loss of hair cells might trigger changes in supporting cells in which the Notch pathway was wholly or partly re-engaged. Indeed, a recent study suggest that treatment of the noise-damaged adult cochlea with gamma secretase inhibitors promoted some trans-differentiation of supporting cells into hair cells [70]. It remains to be determined conclusively whether this effect is specific to the Notch pathway, and whether hair cell loss is necessary for this response to occur. It is also notable that adult vestibular supporting cells are capable of expressing at least some markers of hair cells after gamma secretase treatment [71, 72]. It will therefore be profitable to compare the transcriptomes of supporting cells from different vestibular organs to their cochlear counterparts at different ages, and to compare the epigenetic state of hair cell loci in these different populations of supporting cells as a first step to understand the molecular basis for the different propensities of these cells for trans-differentiation into hair cells.

## Supporting Information

S1 Fig Analysis of P0 and P5 supporting cells response to Notch inhibition. (A): Surface preps of LfngGFP+ cochlear explants. Sensory epithelium retains its GFP expression after culture for 24 hours in either the gamma secretase inhibitor DAPT or DMSO vehicle control. (B): Diagram of the supporting cells recognized from the top of the epithelium. (C): Diagram of the experimental design. P0 or P5 cochleas were dissected and cultured. Explants were then dissociated and sorted for GFP fluorescence and the GFP+ and GFP- fractions used to make RNA-seq libraries. (D): Summary of differentially expressed transcript comparisons in each experimental condition.
(TIF)

S1 Table PCR primers used to generate DNA templates for the synthesis of probes for in situ hybridization. For each gene, forward and reverse PCR primers are given, together with the predicted size of the band generated from PCR with mouse genomic DNA. All reverse primers contain a T7 polymerase sequence at their 5’ end (GGATCCTAATACGACTCACTATAGGGAG).
(XLSX)

S2 Table The entire processed transcriptome for P1 and P6 sorted Lfng-GFP cells. Sheet 1 shows the analysis performed on the data with DESeq for both P1 and P6 cells. Sheet 2 shows the analysis performed with Cufflinks on P1 cells and Sheet 3 shows the same Cufflinks analysis on P6 cells.
(XLSX)

S3 Table The entire processed transcriptome for sorted Lfng-GFP cells from P0 and P5 cochleas cultured in DMSO or DAPT. Sheet 1 shows the analysis performed on the data with DESeq for both P0 and P5 cells. Sheet 2 shows the analysis performed with Cufflinks on P0 cells and Sheet 3 shows the same Cufflinks analysis on P5 cells.
(XLSX)

S4 Table Sample list of known supporting cell genes whose transcripts are enriched in either P1 or P6 Lfng-GFP+ supporting cells. The gene name is indicated, together with the expression level (reads per kilobase of transcript per million mapped reads; RPKM; DESeq output only) and its fold change compared to GFP- cells. p-adj = adjusted p-value for the difference between GFP+ and GFP- populations.
(DOCX)

S5 Table P1 and P6 consensus lists of supporting cells genes. Consensus lists of genes enriched in FACS sorted Lfng-GFP+ cells from postnatal day 1 (P1; 1884 genes) and postnatal day 6 (P6; 1278 genes) mouse cochlea compared to Lfng-GFP-negative cells. Analysis of the sequencing reads was performed by two different approaches. (1) Reads were mapped to the Mus musculus NCBI build37.2 iGenome (Ilumina) using TopHat 2.0 software (Trapnell et al., 2009; Trapnell et al., 2012) and the mapped reads were quantitated and compared using Cufflinks 2.0 providing differential gene expression data and statistics. (2) Reads were aligned to the Mus musculus Ensembl mm9 iGenome (Ilumina) using TopHat 1.4.1 software and the number of reads per gene and per library was obtained using DESeq program. After comparing the level of expression of each gene within each pair of related libraries (GFP+ versus GFP- for P1 and P6 cells), the most significant differentially expressed genes (DEG) were annotated and analyzed separately for both approaches. A consensus list of DEGs common to both methods of analysis was then generated. A significantly DEG was considered to have an RPKM higher than 3000, Fold Change (FC) higher than 4 and p value and FDR < 0.01. Duplicate samples of Lfng-GFP+ and GFP- sorted cells were prepared for P1 and P6. Approximately 60,000 sorted cells were as starting material to generate approximately 100–600 ng RNA (measured by Nanodrop spectrophotometer). cDNA libraries for RNAseq were generated using RNA Seq Truseq RNA sample preparation kit v2 (Illumina) following the low sample protocol for RNA extraction, cDNA synthesis, indexing and amplification. The quality and integrity of RNA samples and the final quality of the sequencing libraries was checked by electrophenogram in an Agilent Bioanalyzer. Paired-end sequencing was performed in HiSeq2000 sequencing platform (Illumina). Fastq files of paired end reads have been deposited in the NCBI GEO database, Accession No. GSE83357.
(XLS)

S6 Table P1 versus P6 LfngGFP+ consensus list of DEG. Consensus list of genes enriched in Lfng-GFP+ supporting cells that were differentially expressed between P1 and P6. Data was obtained from the analysis described in S5 Table caption above, but now genes enriched in supporting cells were compared for changes between P1 and P6.
(XLS)

S7 Table Summary of supporting cell gene candidates validated by in situ hybridization. For each gene, its expression at P1 and P6 (RPKM) together with the fold enrichment between GFP+ and GFP- cell populations is shown, together with expression pattern in the cochlea (SC, supporting cell; HC, hair cell; GER, greater epithelial ridge; SV, stria vascularis; Ubi, ubiquitous expression; No, no detectable signal; ND = not determined). NS: p>0.01.
(DOCX)

S8 Table Summary of transcriptional changes after DAPT treatment of P0 cochlear organ cultures. P0 Lfng-GFP cochlear cultures were maintained for 24 hours in DAPT or DMSO, followed by FACS sorting for GFP fluorescence. DESeq was used to identify transcripts enriched in cultures treated with either DAPT or DMSO. The table shows a list of 2088 genes enriched in Lfng-GFP+ cells that were significantly altered in DAPT-treated cultures compared to DMSO controls. Up-regulated transcripts after DAPT treatment are highlighted in red; down-regulated transcripts are highlighted in green. Duplicate samples of LfngGFP+ and GFP- sorted cells were prepared for postnatal day 0 (P0) and postnatal day 5 (P5) cultured for 24 hours in DMSO or DAPT (10uM). 10,000–20,000 sorted cells were used as starting material to generate approximately 100–600 ng RNA (measured by Nanodrop spectrophotometer). cDNA received an initial amplification using the NuGen Ovation Kit. cDNA libraries for RNAseq were generated using RNA Seq Truseq RNA sample preparation kit v2 (Illumina) following the low sample protocol for RNA extraction, cDNA synthesis, indexing and amplification. The quality and integrity of RNA samples and the final quality of the sequencing libraries was checked by electrophenogram in an Agilent Bioanalyzer. Paired-end sequencing was performed in HiSeq2000 sequencing platform (Illumina). Fastq files of paired end reads have been deposited in the NCBI GEO database, Accession No. GSE83357. Reads were aligned to the Mus musculus Ensembl mm9 iGenome (Ilumina) using TopHat 1.4.1 software and the number of reads per gene and per library was obtained using DESeq program. After comparing the level of expression of each gene within each pair of related libraries, the most significant differentially expressed genes (DEG) were annotated and analyzed separately for both approaches. In order to find genes significantly changing in isolated Lfng-EGFP+ cells obtained from cultured explants (treated or not with DAPT), the level of DEG significance was a FC higher than 2 and a p value and FDR < 0.01.
(XLS)
