# Supplementary figures and images for: Transcriptomic Analysis of Mouse Cochlear Supporting Cell Maturation Reveals Large-Scale Changes in Notch Responsiveness Prior to the Onset of Hearing
Source: PLoS One. 2016 Dec 5;11(12):e0167286. doi: 10.1371/journal.pone.0167286 (PMC5137903; doi:10.1371/journal.pone.0167286)

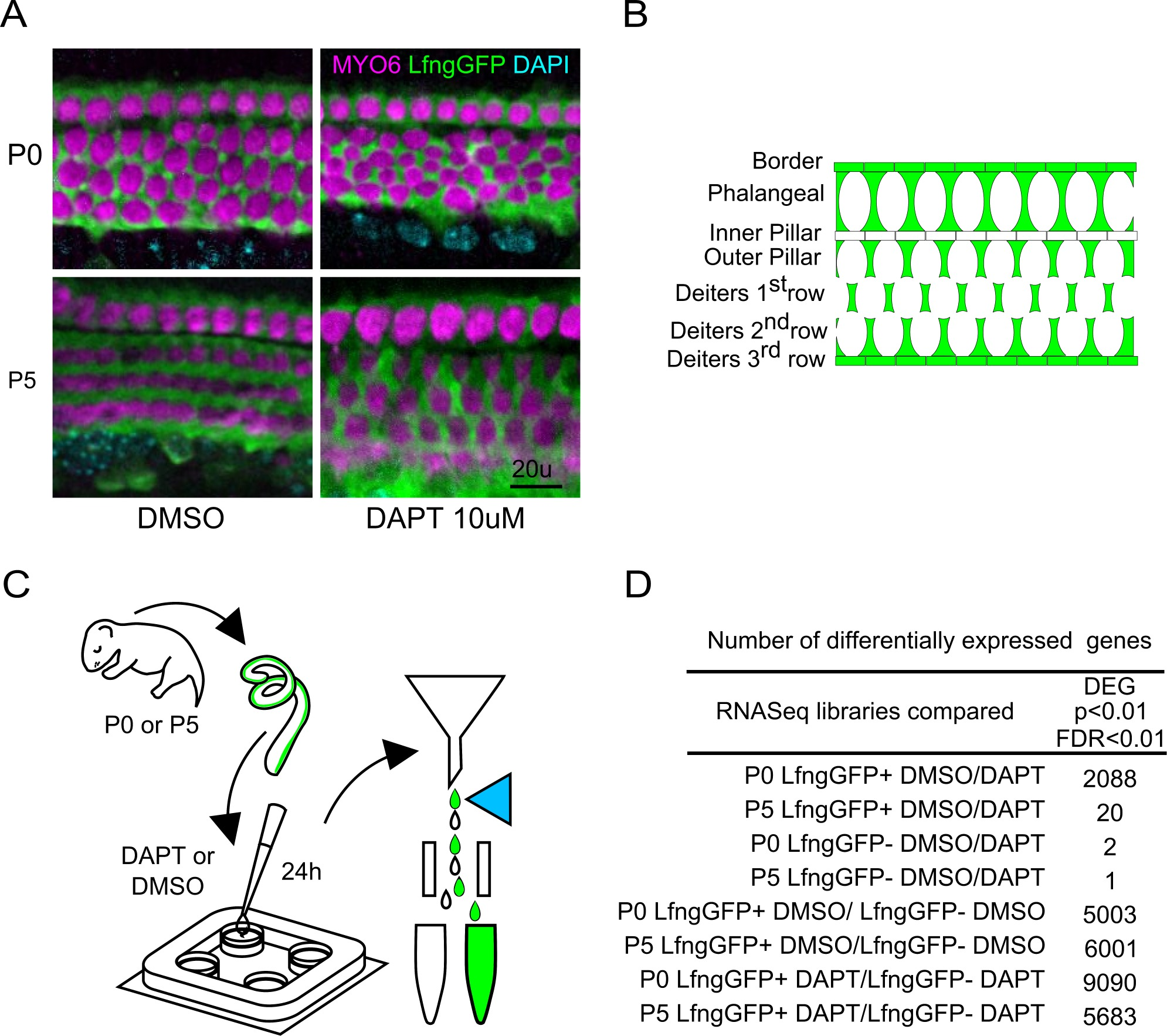

Supplement: S1 Fig — (A): Surface preps of LfngGFP+ cochlear explants. Sensory epithelium retains its GFP expression after culture for 24 hours in either the gamma secretase inhibitor DAPT or DMSO vehicle control. (B): Diagram of the supporting cells recognized from the top of the epithelium. (C): Diagram of the experimental design. P0 or P5 cochleas were dissected and cultured. Explants were then dissociated and sorted for GFP fluorescence and the GFP+ and GFP- fractions used to make RNA-seq libraries. (D): Summary of differentially expressed transcript comparisons in each experimental condition. (TIF) [file pone.0167286.s001.tif]
