# Supplementary material for: Transcriptomic Analysis of Mouse Cochlear Supporting Cell Maturation Reveals Large-Scale Changes in Notch Responsiveness Prior to the Onset of Hearing
Source: PLoS One. 2016 Dec 5;11(12):e0167286. doi: 10.1371/journal.pone.0167286 (PMC5137903; doi:10.1371/journal.pone.0167286)
Supplement: S4 Table — The gene name is indicated, together with the expression level (reads per kilobase of transcript per million mapped reads; RPKM; DESeq output only) and its fold change compared to GFP- cells. p-adj = adjusted p-value for the difference between GFP+ and GFP- populations. (DOCX) [file pone.0167286.s005.docx]

**S4 Table: Previously described supporting cell genes expressed in Lfng-EGFP+ cells during the first postnatal week**

|  | ***Postnatal Day 1*** | | | ***Postnatal Day 6*** | | |  |
| --- | --- | --- | --- | --- | --- | --- | --- |
| **Supporting Cell Genes** | **Expression in Lfng-GFP^+^ (RPKM)** | **Fold change (GFP^+^vs GFP^-^)** | ***p-*adj** | **Expression in Lfng-GFP^+^ (RPKM)** | **Fold change (GFP^+^vs GFP^-^)** | ***p-*adj** | **References** |
| *Hes5* | 6663.27 | 306.73 | 1.68E-16 | 737.46 | 174.97 | 4.39E-24 | Zine and de Ribaupierre, 2002 |
| *Lfng* | 109921.44 | 112.4 | 2.50E-252 | 117586.92 | 81.79 | 2.31E-10 | Zhang et al., 2000 |
| *Fgfr3* | 86721.79 | 92.49 | 1.44E-275 | 49608.94 | 33.2 | 1.18E-18 | Mueller et al., 2002 |
| *Prox1* | 22603.65 | 43.67 | 2.53E-144 | 4088.87 | 32.69 | 4.72E-09 | Bermingham-McDonogh et al., 2006 |
| *Hey1* | 22093.29 | 25.21 | 6.75E-160 | 9892.38 | 4.35 | 0.000014 | Hayashi et al., 2008; Li et al., 2008; Doetzlhofer et al., 2009 |
| *Sox2* | 25105.72 | 22.85 | 5.82E-153 | 12351.66 | 8.46 | 7.83E-12 | Kiernan et al., 2005b; Oesterle et al., 2008 |
| *Jag1* | 81967.23 | 19.58 | 2.12E-105 | 47865.16 | 17.31 | 9.94E-43 | Zine et al., 2000 |
| *Sox21* | 7762.45 | 16.88 | 3.81E-116 | 3997.84 | 10.94 | 4.98E-08 | Hosoya et al., 2011 |
| *Hey2* | 7358.57 | 12.92 | 1.09E-102 | 6735.09 | 10.27 | 1.79E-24 | Hayashi et al., 2008; Li et al., 2008; Doetzlhofer et al., 2009 |
| *Lgr5* | 18883.92 | 12.13 | 1.92E-20 | 11902.63 | 9.7 | 7.56E-28 | Chai et al., 2011 |
| *Cdkn1b* | 31099.06 | 8.34 | 8.20E-83 | 22405.84 | 5.6 | 4.48E-17 | Chen and Segil, 1999; Lowenheim et al., 1999 |
| *Heyl* | 10975.11 | 5.36 | 1.29E-52 | 17507.57 | 14.06 | 3.49E-37 | Hayashi et al., 2008; Li et al., 2008; Doetzlhofer et al., 2009 |
| *Notch1* | 47252.67 | 4.36 | 1.00E-44 | 14809.73 | 1.650383 | 0.208748 | Murata et al., 2006 |
| *Aqp4* | 741.23 | 35.66 | 5.89E-69 | 452.4991 | 1.673351 | 0.165892 | Huang et al., 2002 |
| *Ngfr* | 1481.601 | 1.201585 | 1 | 2659.19 | 6.81 | 2.83E-18 | von Bartheld et al., 1991 |
