# Supplementary material for: Transcriptomic Analysis of Mouse Cochlear Supporting Cell Maturation Reveals Large-Scale Changes in Notch Responsiveness Prior to the Onset of Hearing
Source: PLoS One. 2016 Dec 5;11(12):e0167286. doi: 10.1371/journal.pone.0167286 (PMC5137903; doi:10.1371/journal.pone.0167286)
Supplement: S7 Table — For each gene, its expression at P1 and P6 (RPKM) together with the fold enrichment between GFP+ and GFP- cell populations is shown, together with expression pattern in the cochlea (SC, supporting cell; HC, hair cell; GER, greater epithelial ridge; SV, stria vascularis; Ubi, ubiquitous expression; No, no detectable signal; ND = not determined). NS: p>0.01. (DOCX) [file pone.0167286.s008.docx]

**S7 Table: In situ validation of supporting cell gene candidates at P1 and P6**

| **Gene** | **P1 Cell Type Expression** | **P1 GFP+ Expression**  **(RPKM)** | **P1 Fold Change** | **P6 Cell Type Expression** | **P6 GFP+ Expression**  **(RPKM)** | **P6 Fold Change** |
| --- | --- | --- | --- | --- | --- | --- |
| *Anxa5* | SC, SV | 41188.14 | 7.20 | GER, SV | 57073.61 | 7.25 |
| *B4galnt3* | SC, GER | 36412.04 | 14.34 | SC, GER | 26426.04 | 13.51 |
| *Crhr1* | Ubi | 969.60 | 67.68 | SC | 10718.38 | 310.72 |
| *Daam2* | SC | 48050.49 | 10.60 | No | 7690.45 | 0.98^NS^ |
| *Gm5887* | Ubi | 7392.25 | 154.81 | SC | 17389.04 | 850.84 |
| *Gpc1* | SC, GER, LER | 46693.28 | 9.00 | No | 10114.52 | 1.78^NS^ |
| *Igfbp3* | SC, GER | 82193.80 | 21.75 | ND | 70128.52 | 18.25 |
| *Igfbp5* | SC, GER, LER | 387077.54 | 4.07 | ND | 199707.92 | 1.57^NS^ |
| *Inhba* | SC | 4837.93 | 100.20 | ND | 1690.11 | 15.01 |
| *Rassf6* | Ubi | 2012.60 | 19.07 | SC | 7298.55 | 77.97 |
| *Raver2* | SC, HC | 36964.09 | 14.77 | No | 4570.41 | 3.08^NS^ |
| *Sapcd2* | Ubi | 4046.79 | 11.92 | SC | 15279.43 | 50.42 |
| *Skp1a* | SC, LER | 62393.42 | 6.51 | SC, GER, LER | 142556.98 | 7.80 |
| *Slitrk6* | SC | 47604.15 | 120.46 | No | 35908.27 | 36.59 |
| *Tgfb2* | SC, GER | 44591.12 | 7.07 | ND | 15425.88 | 1.97^NS^ |
| *Tmprss3* | SC, HC | 14849.67 | 50.63 | SC | 15912.06 | 42.17 |
| *Tppp* | Ubi | 2370.07 | 11.11 | SC | 4647.09 | 41.33 |
| *Tsga14* | SC | 39687.92 | 49.87 | SC | 79752.75 | 139.51 |
| *Ttyh1* | SC, GER | 38270.96 | 9.35 | No | 17669.90 | 9.14 |
| *Uchl1* | SC | 25282.67 | 10.05 | SC | 15780.27 | 14.23 |
| *Wnt7a* | Ubi | 10897.69 | 46.84 | SC | 7143.40 | 125.77 |
